# Care of the Finger Nails

**Published:** 1892-09

**Authors:** 


### THE CARE OF FINGER NAILS.

These require special attention if we desire to preserve them in their highest condition of beauty and usefulness. To keep them clean, the nall brush and soap and water should be used once or oftener daily, as circumstances demand. Once a day at least in wiping the hands after washing them, and, while they are still soft from the action of the water, the free edge of the scarf skin, which, if not attended to, is apt to grow upward over the nails, should be loosened and pressed back in a neatly rounded form, by which the occurrence of cracks and sores about their roots (agnails, nailsprings, etc.) will be prevented, and a graceful oval form, ending in a crescent-like space of white, will be insured. This skin, as a rule, should never be cut, pared, picked, or torn off, as is commonly done, and the less it is meddled with, otherwise than in the way just mentioned, the better.

